# Miss Lathrop Points to New Zealand as Model

**Published:** 1919-02

**Authors:** 


### Miss Lathrop Points to New Zealand as Model.

For a considerable term of years, Julia Lathrop says, New Zealand has secured and maintained lower infant mortality rates than those recorded for any other country. For the last nine years this country has prosecuted a vigorous educational campaign carried on by the Government and by the New Zealand Society for the help of women and children, Clinton Rogers Woodruff writes in the Living Church.

The society describes itself by its name as being one for mutual helpfulness and mutual education in the field. Its chief aims are (1) to uphold the sacredness of the body and the duty of health; (2) to acquire and disseminate accurate information and knowledge on matters affecting the health of women and children; (3) to train specially and to employ qualified nurses whose duty it will be to give gratis, to any member of the community desiring such services, sound, reliable instruction, advice and assistance on matters affecting the health and well-being of women and their children.

While the effects of the war thus far are scarcely appreciable so far as infant mortality in the United States is concerned, America may well take warning, according to Dr. Franklin Martin, chairman of the Advisory Commission of the Council on National Defense, and give consideration to the experience of France and to a less alarming degree to the other nations that have been at war for nearly four years. The birth rate of all warring countries is necessaryily less than in normal times.

